# Draft Genome Sequence of an Extended-Spectrum-β-Lactamase-Positive Hypervirulent *Klebsiella pneumoniae* Strain with Novel Sequence Type 2318 Isolated from a Neonate

**DOI:** 10.1128/genomeA.01273-16

**Published:** 2016-11-10

**Authors:** Chaitra Shankar, Sridhar Santhanam, Manish Kumar, Vijay Gupta, Naveen Kumar Devanga Ragupathi, Balaji Veeraraghavan

**Affiliations:** a Department of Clinical Microbiology, Christian Medical College, Vellore, Tamil Nadu, India; b Department of Neonatology, Christian Medical College, Vellore, Tamil Nadu, India

## Abstract

Antimicrobial resistance among hypervirulent *Klebsiella pneumoniae* is increasingly reported. Here, we report the draft genome sequence of a hypervirulent *K. pneumoniae* strain isolated from a neonate with sepsis belonging to novel sequence type 2318 (ST2318).

## GENOME ANNOUNCEMENT

*Klebsiella pneumoniae* has recently become a prominent infectious agent among adults and neonates, with an increase in antimicrobial resistance and virulence factors. The hypervirulent (hv) strains which are phenotypically detected by a string test are associated with many virulence genes, such as *rmpA*, *rmpA2,* and those encoding siderophores (1). Biofilm formation is another important virulence factor in bacteremic isolates containing the *mrkD* gene (2).

The isolate in the present study was obtained from a baby girl with a birth weight of 1,500 g and who was born preterm. She was brought to a community hospital on day 3 of life with lethargy and inability to feed and was then referred to our hospital. Initially, the baby had hepatosplenomegaly with severe thrombocytopenia, coagulopathy, and hypotension/metabolic acidosis. She received one fresh frozen plasma transfusion and two platelet transfusions. She was on inotropes for four days. She was started on cefoperazone-sulbactam and amikacin because of suspicion of sepsis. The initial blood culture showed no growth of bacteria. She improved clinically, but thrombocytopenia persisted and her C-reactive protein (CRP) level was high. Hence, blood culture was repeated on day 10 of life and grew extended-spectrum-β-lactamase (ESBL)-positive *K. pneumoniae*. Her antibiotics were changed to meropenem with amikacin.

The baby was readmitted after discharge at 30 days of life with sepsis-like illness and again at 45 days of life with sepsis and feed intolerance treated as suspected necrotizing enterocolitis. She was discharged on both occasions after conservative management.

The isolate was subjected to a string test to identify it as probable hv *K. pneumoniae*. DNA was extracted using QIAsymphony. Whole-genome sequencing was performed by next-generation sequencing using Ion Torrent and assembled using SPAdes version 5.0. The contigs were annotated using RAST (http://rast.nmpdr.org/) and Patric (http://patricbrc.org/). The multilocus sequence typing (MLST), ResFinder (https://cge.cbs.dtu.dk/services/ResFinder/), and PlasmidFinder (https://cge.cbs.dtu.dk/services/PlasmidFinder/) databases were used to find the sequence type, antibiotic resistance genes, and plasmid types present in the isolates. Virulence genes were defined with the help of the database available at http://bigsdb.pasteur.fr/klebsiella/.

Hypervirulent strains characteristically harbor the *rmpA* gene and its variant *rmpA2*, which are regulators of the mucoid phenotype (1). They are often associated with K1 and K2 capsular types. hv *K. pneumoniae* of the K1 capsular type often belongs to clonal complex 23 (CC23) (3, 4), and strains of the K2 type are associated with sequence type 86 (ST86), ST375, and ST380 (3, 5). *rmpA* and *rmpA2* were present in our study isolate. PCR was performed for the detection of *magA* corresponding to the K1 type, and the *k2A* gene for the K2 type (6). However, this isolate belonged to neither the K1 or K2 serotype nor to the clonal types reported. It belonged to a novel type, ST2318. The results from PlasmidFinder showed that it lacked plasmids. Among the first- and second-line antimicrobials tested, it was resistant to cefpodoxime and carried *bla*_SHV-11_,* oqxA*, and *oqxB*. Hypervirulent strains were most often susceptible to first- and second-line antibiotics (7, 8), but recently, reports of multidrug-resistant (MDR)-hv *K. pneumoniae* strains have been on the rise (9). The isolate also coded for siderophores, such as aerobactin, enterobactin, and yersiniabactin. *ybbW* and *allR*, coding for allantoin metabolism, were present. *iroN*, coding for a siderophore receptor, and *mrkD*, coding for type 3 fimbriae, were present.

### Accession number(s).

The whole-genome sequence of the isolate has been deposited at GenBank under accession number LYYE00000000.
